# Volatiles of different resistant cotton varieties mediate the host preference of Mirid bug *Apolygus lucorum*

**DOI:** 10.3389/fpls.2024.1428234

**Published:** 2024-06-12

**Authors:** Juan Wu, Yang Cao, Dong Teng, Shuang Shan, Ting Geng, Xinzheng Huang, Yongjun Zhang

**Affiliations:** ^1^ School of Agriculture and Life Science, Shanxi Datong University, Datong, China; ^2^ Institute of Tropical Bioscience and Biotechnology, Chinese Academy of Tropical Agricultural Sciences, Haikou, China; ^3^ State Key Laboratory for Biology of Plant Diseases and Insect Pests, Institute of Plant Protection, Chinese Academy of Agricultural Sciences, Beijing, China; ^4^ State Key Laboratory of Resource Insects, Institute of Apicultural Research, Chinese Academy of Agricultural Sciences, Beijing, China; ^5^ National Plant Protection Scientific Observation and Experiment Station, Chinese Academy of Agricultural Sciences, Langfang, China; ^6^ Department of Entomology, College of Plant Protection, China Agricultural University, Beijing, China; ^7^ Zhongyuan Research Center, Chinese Academy of Agricultural Sciences, Xinxiang, China

**Keywords:** cotton, mirid bug, plant volatiles, electrophysiological responses, behavioral preference

## Abstract

Cotton, a crucial economic crop, is also the preferred host plant of the mirid bug *Apolygus lucorum*. In our previous field experiments, we found that cotton cultivars Kelin 08–15 and BR-S-10 (healthy and herbivore-damaged plants) exhibit distinct attraction and repellence to *A. lucorum*, respectively. However, the key plant volatiles determining attraction or repulsion effects remain unknown. Here, we investigated the volatiles emitted by these two cotton cultivars before and after herbivore infestation. We found that susceptible Kelin 08–15 emitted a greater diversity and quantity of volatiles than those of BR-S-10, with herbivore-damaged cottons releasing more volatile substances. Electroantennogram (EAG) recordings further revealed that 15 representative volatiles identified above could elicited electrophysiological responses in female and male *A. lucorum* antennae. Among them, behavioral assays showed that two compounds, 1,3-Diethylbenzene and 4-Ethylbenzaldehyde, exhibited attractive properties, whereas six volatiles including Hexyl Acrylate, Cumene, 2,4-Dimethylstyrene, Eucalyptol, Linalool and Butyl Acrylate demonstrated repellent effects on *A. lucorum*. Taken together, our findings suggest the critical role of volatile compounds in mediating bug-plant interactions and provide a foundation for the development of strategies to prevent and control of *A. lucorum* in cotton fields.

## 1 Introduction

The growth and development of cotton plants are threatened by a multitude of pests, resulting in significant yield losses and economic damages worldwide (Wu and Guo, 2005; Luttrell et al., 2015). The commercialization of *Bacillus thuringiensis* (Bt) transgenic cotton has led to effective control of lepidopteran insects, including cotton bollworm (Malaquias et al., 2021). However, the dramatic increase of *Apolygus lucorum* populations in cotton fields has resulted in substantial economic losses annually (Jiang et al., 2015). In response to the resurgence of this notorious pest, researchers have refocused their attention on developing strategies for *A. lucorum* management (Lu et al., 2010). As a polyphagous pest, *A. lucorum* can thrive on diverse hosts, exploiting its highly developed chemosensory system to switch host plants in the field, thereby causing significant damage to multiple crops, including cotton (Pan et al., 2013). The excessive use of insecticides to control *A. lucorum* has been associated with rapid development of insect resistance, pesticide residues, and non-target organism mortality (Zhang et al., 2020; Hua et al., 2023). Therefore, it is crucial to prioritize sustainable and environmentally friendly approaches to efficiently manage *A. lucorum* populations and mitigate the associated ecological and economic impacts.

Exploiting host plant resistance to insects has been reported as an eco-friendly and effective method for pest control, serving as a viable alternative to insecticides (Broekgaarden et al., 2011). Cotton varieties exhibit varying levels of resistance to insect feeding, with cotton Kelin 08–15 being a susceptible variety and cotton BR-S-10 being a resistant variety that can avoid bug feeding (Cao et al., 2012). As part of their biotic stress response, resistant plants possess diverse defense strategies to perceive and endure stress from insects or pathogens by producing a wide range of chemical constituents, including allelochemicals (Tosh et al., 2003; Dixit et al., 2020). Among these constituents, volatile secondary metabolites play a crucial role in host finding, feeding, mating, and oviposition for herbivorous insects (Dicke, 2016). For instance, a transgenic rice line expressing the terpenoid synthase gene *OsTPS46* showed increased resistance to *Rhopalosiphum padi* due to the emission of specific volatile compounds, including lemonene, (*E*)-β-farnesene, and linalool (Sun et al., 2017). Similarly, a resistant *Arabidopsis* line with high emissions of (3*E*)-4,8-dimethyl-1,3,7-nonatriene (DMNT) repelled *Plutella xylostella* and reduced the survival rate of larvae feeding on the resistant line (Chen et al., 2021a). Herbivore-induced stress can stimulate significant changes in plant volatile production, which tend to possess insect-resistant functions (Wani et al., 2022). For example, DMNT emitted by *Spodoptera littoralis*-infested cotton affected its tendency choice, thereby influencing mating and oviposition behavior (Hatano et al., 2015). Similarly, *Spodoptera exigua*-injured corn released (*Z*)-3-hexenyl acetate, which triggered plant defense against herbivorous insects (Engelberth et al., 2004). However, it remains unclear whether there are differences in the volatile profiles of susceptible cotton Kelin 08–15 and resistant cotton BR-S-10 before and after herbivore-damaged stress. Are these differential volatiles related to insect resistance?

In the present study, we employed a dynamic headspace sampling system coupled with gas chromatography-mass spectrometry (GC-MS) to collect and analyze plant volatiles from resistant and susceptible cotton varieties. Moreover, the significant increase in sap-sucking insects’ population has had a profound impact on cotton yields, such as *A. lucorum*. Therefore, we also collected volatiles from cotton plants damaged by *A. lucorum*. The volatile profiles of different treatments, including resistant and susceptible cotton varieties, herbivore-damaged and undamaged cotton plants, were determined and compared. We identified differential volatiles and further investigated the electrophysiological and behavioral responses of *A. lucorum* adults to these volatiles using electroantennogram (EAG) recordings and Y-tube olfactometer assays, respectively. The outcomes of this study will contribute to the identification of cotton volatile compounds with potential ecological functions, which can inform the development of promising and sustainable approaches to enhance biological control strategies.

## 2 Materials and methods

### 2.1 Plants and insects

Cotton cultivars BR-S-10 (resistant) and Kelin 08–15 (susceptible) were obtained from the Xinxiang Plant Protection Scientific Observation and Experiment Station of the Chinese Academy of Agricultural Sciences, Henan Province, China (35°09′ N, 113°48′ E). The cotton plants were grown in plastic pots (15 cm in height and diameter) in a greenhouse with a temperature of 27 ± 2°C, relative humidity of 50 ± 10%, and a 16:8 light/dark cycle. Water was supplied every two days for irrigation. Cotton plants with 6~7 fully expanded leaves were used in next experiment.

Three days old adults of *A. lucorum* were kindly provided by the National Plant Protection Scientific Observation and Experiment Station Langfang, Hebei Province, China (39°08′ N, 116°23′ E). The laboratory colony was maintained at a temperature of 28 ± 1°C, relative humidity of 60 ± 10%, and a 14:10 h light/dark photoperiod.

### 2.2 Plant treatment

To investigate the herbivore-induced plant volatiles (HIPVs) emitted from resistant cotton cultivar BR-S-10 and susceptible cotton cultivar Kelin 08–15, ten *A. lucorum* adults were placed on each cotton plant. After 48 hours of exposure, the bugs were carefully removed, and the cotton plants were immediately subjected to subsequent HIPVs collection. Cotton plants without herbivore damage maintained under identical conditions were served as controls. The experiment was conducted with three biological replicates for each treatment.

### 2.3 Volatile collection and analysis

Plant volatiles emitted from herbivore-damaged and healthy cotton plants were collected using a headspace sampling system (Huang et al., 2015). Briefly, pots containing one *A. lucorum*-damaged or control plant were placed within a glass jar (30 cm in height and diameter) equipped with inlet and outlet ports. A vacuum pump was used to draw air through the jar at a flow rate of 1 L/min. The volatiles were then trapped on 50 mg of 60/80 mesh Tenax-TA (Shanghai ANPEL Scientific Instrument Company, Shanghai, China) in an 8-mm-diameter glass cartridge located at the outlet. Volatile sampling was conducted over a 4-hour period. Following collection, the volatiles were extracted with 200 μl of dichloromethane, to which n-octane (Sigma-Aldrich) was added as an internal standard for quantitative analysis. This procedure was repeated three times for each treatment.

The extracted volatile samples were then analyzed using a Shimadzu GC-MS system (GC6890-MSD5973, Agilent Technologies, CA) equipped with a HP-5MS column (30 m × 0.25 mm × 0.25 μm, Agilent Technologies, CA). The GC oven temperature was programmed as follows: initial temperature of 40°C for 1 min, increasing to 100°C at a rate of 4°C/min (held for 1 min), then to 150°C at a rate of 6°C/min (held for 1 min), and finally to 250°C at a rate of 10°C/min (held for 5 min). Peak identification was performed by comparing retention times and mass spectra with those of authentic standards analyzed under the same conditions.

### 2.4 Electroantennogram recordings

Based on the GC-MS results, 15 volatile compounds were selected for EAG recordings to evaluate their effects on the antennal responses of *A. lucorum* adults. These compounds included Hexyl Acrylate, Cumene, 1,3-Diethylbenzene, 1,4-Diethylbenzene, 2,4-Dimethylstyrene, 4-Ethylbenzaldehyde, Naphthalene, 4-Ethylacetophenone, 2,2,4,4,6,8,8-Heptamethylnonane, P-Xylene, (1R)-(+)-α-Pinene, Camphene, Eucalyptol, Linalool, and Butyl Acrylate. In EAG recordings, standard compounds were dissolved in liquid paraffin at a concentration of 100 μg/μL. Pure liquid paraffin and methyl phenylacetate were used as blank control and reference compound, respectively. A piece of folded filter paper (5 mm × 20 mm) with 50 μL of each compound was placed into a glass Pasteur pipet. The antenna of 3-day-old adult bug was carefully removed at the base and tip, and then instantly connected to electrode holders using electrode gel. The stimulus was delivered to the antennal sample through a constant flow of clean air (activated charcoal-filtered and humidified) at a rate of 300 mL/min for 0.3 s. EAG signals were recorded and analyzed using a Syntech IDAC-2 (Intelligent Data Acquisition Controller) and EAGPro V. 2 (Syntech, Kirchzarten, Germany). Each compound was tested on 15 antennal samples.

### 2.5 Behavioral assays

Based on the results of volatile analysis and EAG recordings, compounds identified as eliciting strong EAG responses in *A. lucorum* adults were used in subsequent behavioral assays. The behavioral responses of *A. lucorum* to volatile standards were evaluated in a Y-tube olfactometer. The olfactometer consisted of a 30 cm main stem, two 30 cm lateral arms, and a 60° angle between arms, all made of glass. The two branch tubes were connected to separate odor-source flasks. The Y-tube was placed into a steel chamber (1 m × 0.8 m × 0.8 m) which was equipped with two 40-W fluorescent lamps providing uniform lighting (~2000 lux) as illumination sources. Each compound was individually formulated in liquid paraffin to a concentration of 100 μg/μL, and a 50 µL sample was applied to a filter paper strip (50 mm × 5 mm) before being placed inside the treatment flask. Pure liquid paraffin was served as the control. The stimuli were delivered to the olfactometer arms at a constant air flow rate of 500 mL/min. Three-day-old adult bugs were released individually at the base of the central arm. A bug was considered to have made a choice if it reached the midpoint of the lateral arm and remained there for at least 5 s. If a bug failed to make a choice within 5 min, it was recorded as no choice. Each insect was used only once. After testing five bugs, the position of the treatment and control arms were switched. The Y-tube olfactometer was replaced with a clean one after testing ten individuals. A total of 60 adult bugs were tested for each compound.

### 2.6 Data analysis

All data were analyzed using SPSS STATISTICS 18.0 software (SPSS Inc., Chicago, IL, USA). The results are presented as the mean ± standard error of the mean (SEM). To compare volatile emissions between different treatments, a one-way analysis of variance (ANOVA) was performed, followed by the least-significant difference (LSD) test. The significance level was set at *P* < 0.05. For EAG experiments, the relative EAG value was calculated using the following formula: EAG relative value = [(EAG value of compound - EAG value of control)/(EAG value of reference - EAG value of control)] × 100. Student’s t-test was used to compare recorded EAG values between males and females, with a significance level of *P* < 0.05. In the Y-tube behavioral trial, a Chi-Square test with a 50:50 distribution was performed to determine the preference of bugs between plant volatile standards and controls.

## 3 Results

### 3.1 Volatiles emitted from resistant cotton BR-S-10 and susceptible cotton Kelin 08–15

GC-MS analysis showed significant differences in the volatile profiles of BR-S-10 and Kelin 08–15. Notably, six compounds including Hexyl Acrylate, 2,4-Dimethylstyrene, 4-Ethylbenzaldehyde, m-Ethylacetophenone, 1-(4-Ethylphenyl)ethanone, and 2,2,4,4,6,8,8-Heptamethylnonane, were unique to the volatile emissions of susceptible cotton Kelin 08–15, whereas 1-Methylethylbenzene1) was exclusively released by resistant cotton BR-S-10. The quantitative analysis of each compound was further performed by comparing the peak area ratio to an internal standard (**Table 1**). However, the emissions of 1,3-Diethylbenzene (*P* = 0.0012), 1,4-Diethylbenzene (*P* = 0.0001), and Naphthalene (*P* = 0.0425) were significantly lower in resistant cotton BR-S-10 compared to susceptible cotton Kelin 08–15.

**Table 1:** Differential volatiles collected from BR-S-10 and Kelin 08–15 cotton plants.

Compounds	BR-S-10	Kelin 08–15
Hexyl Acrylate	ND	0.0177 ± 0.0098 *
1-Methylethylbenzene	0.0373 ± 0.0188 *	ND
1,3-Diethylbenzene	0.1894 ± 0.0176	7.9215 ± 0.9446 *
1,4-Diethylbenzene	0.2691 ± 0.0473	6.4335 ± 0.0533 *
2,4-Dimethylstyrene	ND	0.5935 ± 0.0543 *
4-Ethylbenzaldehyde	ND	0.1764 ± 0.0265 *
Naphthalene	0.0466 ± 0.0466	0.2244 ± 0.0386 *
m-Ethylacetophenone	ND	0.8265 ± 0.0559 *
1-(4-Ethylphenyl)ethanone	ND	1.1503 ± 0.0618 *
2,2,4,4,6,8,8-Heptamethylnonane	ND	0.0568 ± 0.0284 *

Amounts (means ± SE) measured in ng/4 hr. ND, not detected. Asterisk indicates significant differences at < 0.05 level.

### 3.2 Volatiles emitted from herbivore-damaged cottons

The composition and content of volatiles emitted from healthy and herbivore-damaged cotton plants also exhibited significant differences. Nine differential volatiles were identified in *A. lucorum*-damaged BR-S-10 plants, with five compounds showing slight up-regulation (Ethylbenzene, p-Xylene, 2-Propenoic acid butyl ester, Camphene and 1,4-Dichlorobenzene) and four compounds showing slight down-regulation (n-Butyl ether, (1*R*)-(+)-α-Pinene, 1,3-Diethylbenzene, and 1,4-Diethylbenzene). Two compounds including 1-Methylethylbenzene, and Eucalyptol were not detected (**Table 2**).

**Table 2:** Volatiles emitted from undamaged and herbivore-damaged BR-S-10 cotton plants.

Compounds	Healthy BR-S-10 plants	*A. lucorum*-damaged BR-S-10 plants
Ethylbenzene	0.0140 ± 0.0140	0.0220 ± 0.0110
p-Xylene	0.0775 ± 0.0661	0.1157 ± 0.0596
n-Butyl ether	0.0331 ± 0.0166	0.0231 ± 0.0159
2-Propenoic acid butyl ester	0.1388 ± 0.0110	0.2112 ± 0.0381
1-Methylethylbenzene	0.0373 ± 0.0188 *	ND
(1*R*)-(+)-α-Pinene	0.0606 ± 0.0231	0.0308 ± 0.0167
Camphene	0.0249 ± 0.0249	0.0290 ± 0.0171
1,4-Dichlorobenzene	0.5743 ± 0.0803	0.6153 ± 0.0178
Eucalyptol	0.0833 ± 0.0833 *	ND
1,3-Diethylbenzene	0.1894 ± 0.0176 *	0.1129 ± 0.0132
1,4-Diethylbenzene	0.2691 ± 0.0473 *	0.1516 ± 0.01649

Amounts (means ± SE) measured in ng/4 hr. ND, not detected. Asterisk indicates significant differences at < 0.05 level.

In *A. lucorum*-damaged cotton cultivar Kelin 08–15, p-Xylene and 2,2,4,4,6,8,8-Heptamethylnonane were not detected, whereas the emissions of (1*R*)-(+)-α-Pinene, 1,3-Diethylbenzene, 1,4-Diethylbenzene, 2,4-Dimethylstyrene, 4-Ethylbenzaldehyde, Naphthalene, m-Ethylacetophenone, and 1-(4-Ethylphenyl) ethanone were increased. Only 1,4-Dichlorobenzene showed decreased emission **Table 3**.

**Table 3:** Volatiles emitted from undamaged and herbivore-damaged Kelin 08–15 cotton plants.

Compounds	Healthy Kelin 08–15 plants	*A. lucorum*-damaged Kelin 08–15 plants
p-Xylene	0.0205 ± 0.0079 *	ND
(1*R*)-(+)-α-Pinene	0.0062 ± 0.0062	0.0618 ± 0.0142 *
1,4-Dichlorobenzene	0.4905 ± 0.0143	0.4079 ± 0.0522
1,3-Diethylbenzene	7.9215 ± 0.9446	10.6594 ± 1.4801
1,4-Diethylbenzene	6.4335 ± 0.0533	6.5257 ± 0.6954
2,4-Dimethylstyrene	0.5935 ± 0.0543	0.8940 ± 0.1335
4-Ethylbenzaldehyde	0.1576 ± 0.0201	0.2487 ± 0.0797
Naphthalene	0.1907 ± 0.0067	0.2200 ± 0.0273
m-Ethylacetophenone	0.8265 ± 0.0559	1.2356 ± 0.1585
1-(4-Ethylphenyl)ethanone	1.1503 ± 0.0618	1.4976 ± 0.1926
2,2,4,4,6,8,8-Heptamethylnonane	0.0568 ± 0.0284 *	ND

Amounts (means ± SE) measured in ng/4 hr. ND, not detected. Asterisk indicates significant differences at < 0.05 level.

### 3.3 EAG recordings

**Figure 1:**
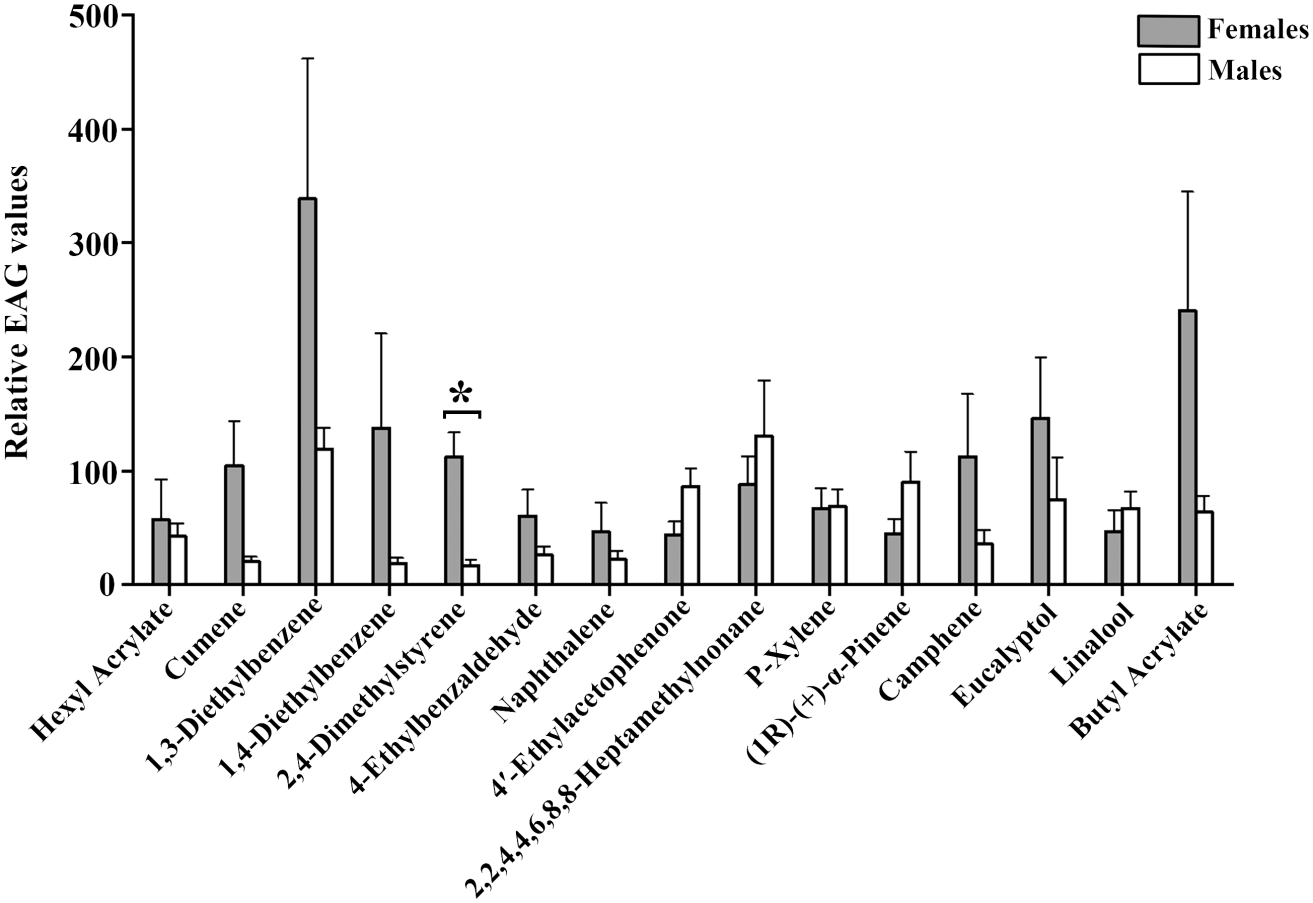
EAG recordings of male and female *A. lucorum* adults to 15 plant volatile compounds. Error bars represent standard error of the mean (SEM). Significant differences between males and females are denoted by asterisks at *P* < 0.05.

Fifteen selected cotton volatile compounds were used to investigate the EAG responses of *A. lucorum* (**Table 4**). All 15 compounds elicited intense EAG responses in both female and male *A. lucorum* adults. Notably, 2,4-Dimethylstyrene exhibited significant sexual dimorphism, with stronger EAG responses in females compared to males. No significant differences in EAG responses to the other compounds were observed between males and females (**Figure 1**).

**Table 4:** Volatile compound standards used in EAG and behavioral assays.

Number	Sample name	Pure degree (%)	Density (g/ml)
1	Hexyl Acrylate	98	0.888
2	Cumene	99.9	0.862
3	1,3-Diethylbenzene	99	0.86
4	1,4-Diethylbenzene	98	0.86
5	2,4-Dimethylstyrene	97	0.904
6	4-Ethylbenzaldehyde	98	0.979
7	Naphthalene	99.5	1.145
8	4-Ethylacetophenone	97	0.993
9	2,2,4,4,6,8,8-Heptamethylnonane	98	0.793
10	P-Xylene	99	0.86
11	(1*R*)-(+)-α-Pinene	98	0.858
12	Camphene	95	Solid
13	Eucalyptol	99.7	0.921
14	Linalool	97	0.86
15	Butyl Acrylate	99.5	0.9015

### 3.4 Behavioral Responses of *A. lucorum* to cotton volatiles

**Figure 2:**
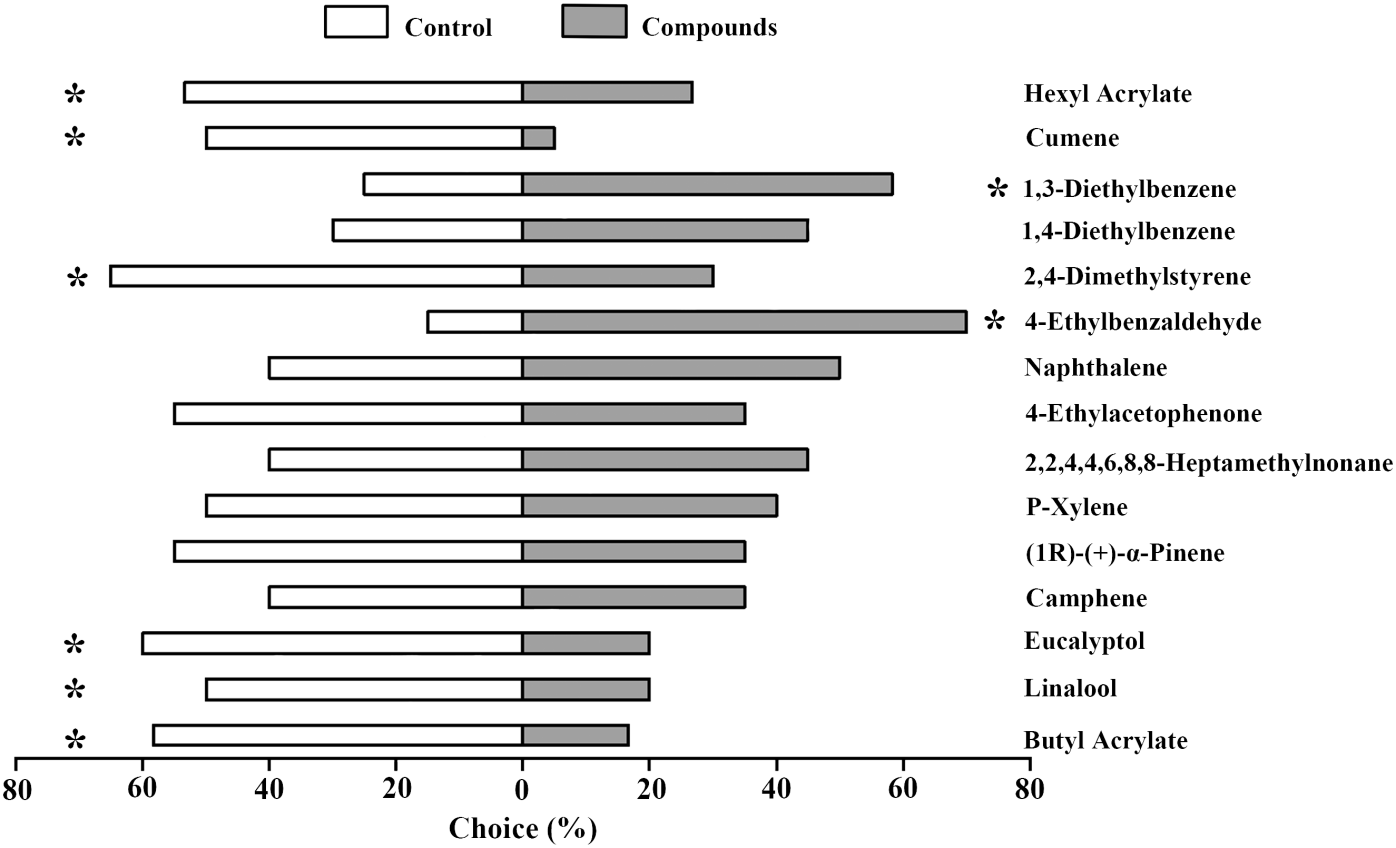
Behavioral responses of *A. lucorum* adults to 15 plant volatile compounds. The proportion of individuals choosing mineral oil (white bars) *versus* compound stimuli (grey bars) is presented. Asterisks indicate significant differences between test and control groups at 0.05 level.

To further investigate the behavioral preference of *A. lucorum* to the 15 selected compounds which could elicit EAG responses in both female and male adults, we performed Y-tube olfactometer assays. Our results indicated that two compounds, 1,3-Diethylbenzene and 4-Ethylbenzaldehyde acting as attractants could induce significant positive behavioral preference of *A. lucorum*. In contrast, six compounds including Hexyl Acrylate, Cumene, 2,4-Dimethylstyrene, Eucalyptol, Linalool and Butyl Acrylate showed significant repellent effects on *A. lucorum*. The remaining seven compounds had no obvious behavioral influence (**Figure 2**).

## 4 Discussion

Cotton plants is an important summer host for the mirid bug, *A. lucorum*. Our previous observation found that *A. lucorum* exhibited differential selection and adaptation to resistant cotton BR-S-10 and susceptible cotton Kelin 08–15. Plants interact with external environment such as other plants, insects and abiotic factors through releasing a diverse array of volatile compounds (Dudareva et al., 2013). For instance, silence of the linalool synthase gene *OsLIS* in rice plants fails to release linalool, consequently, the attractiveness of transegenic rice plants to the brown planthopper, *Nilaparvata lugens*, were increased (Xiao et al., 2012). To investigate the volatile compounds emitted by resistant and sensitive cotton varieties, we performed GC-MS analysis on BR-S-10 and Kelin 08–15. Here, our results revealed significant differences in the volatile profiles between the BR-S-10 and kelin 08–15. Notably, several volatile compounds including Hexyl Acrylate, 2,4-Dimethylstyrene, 4-Ethylbenzaldehyde, m-Ethylacetophenone, 1-(4-Ethylphenyl) ethanone, and 2,2,4,4,6,8,8-Heptamethylnonan were exclusively detected in susceptible cotton Kelin 08–15. Meanwhile, three volatile compounds, 1,3-Diethylbenzene, 1,4-Diethylbenzene and Naphthalene, exhibited higher emission in BR-S-10. These volatile compounds described above are also found in other plant species (Zhang et al., 2019; Koner et al., 2022; de Paula et al., 2023; Qu et al., 2023). For example, 2,4-Dimethylstyrene has been identified as an aroma compound in mango and a key odorant responsible for the chestnut-like aroma of green tea (Zhang et al., 2019; Qu et al., 2023). It is well established that cotton plants damaged by piercing-sucking hemipterans, such as *A. lucorum*, undergo both quantitative and qualitative changes in their volatile emissions (Williams et al., 2005; Huang et al., 2015). Similarly, the emissions of Camphene, (1*R*)-(+)-α-Pinene, 1,3-Diethylbenzene, Eucalyptol, and other volatiles are altered following insect damage. However, in this study, *A. lucorum*-damaged cotton plants emitted fewer types of volatiles than healthy cotton plants. We hypothesize that the response mechanisms of plants to stress may vary across different plant-herbivore interaction systems. Similarly, Isopropyl myristate, (*E*,*E*)-2,4-heptadienal and (*E*,*E*) 2,4-hexadienal emitted in healthy rice, and can’t emitted after being induced by rice planthopper (Chen et al., 2021b). In any case, insect-induced plant volatiles (HIPVs) widespread in plants often play crucial roles in both direct defense and indirect defense (Santos and Rao, 2000; Kessler et al., 2006; Salehi et al., 2019).

To investigate the biological roles of differential volatiles emitted from resistant and susceptible cultivars, we used 15 identified volatile compounds to assess their impacts on the electrophysiological and behavioral response of *A. lucorum*. EAG recordings confirmed that the 15 candidate volatile compounds could elicit strong EAG responses in *A. lucorum*. Additionally, Y-tube assays revealed that *A. lucorum* responded positively to 1,3-Diethylbenzene and 4-Ethylbenzaldehyde. Notably, the emission of 1,3-Diethylbenzene was significantly higher in susceptible cotton Kelin 08–15 than in resistant cotton BR-S-10, while 4-Ethylbenzaldehyde was exclusively released from Kelin 08–15. Interestingly, 1,3-Diethylbenzene has been reported to predominate in the volatile organic compounds of *Rumex dentatus* and is attractive to *Galerucella placida* females (Koner et al., 2022). In contrast, six compounds including 2,4-Dimethylstyrene, Eucalyptol, Linalool and so on exhibited significant repellency against *A. lucorum*. Eucalyptol, a component of essential oil isolated from *Eucalyptus globulus*, *Cinnamomum longepaniculatum*, *Rosmarinus officinalis*, and *Salvia japonica*, has been shown to have repellent activities against *Aedes albopictus* (Huang et al., 2010; Cai et al., 2021). These certain volatiles with attraction or repellence to *A. lucorum* may be used in developing novel pest management strategy. Moreover, resistant cotton BR-S-10 can be used as insect-resistant germplasm resources for biological breeding. These findings provide valuable insights in understanding sustainable and environmentally friendly pest management.

## Data availability statement

The raw data supporting the conclusions of this article will be made available by the authors, without undue reservation.

## Author contributions

JW: Conceptualization, Data curation, Formal analysis, Investigation, Methodology, Writing – original draft, Writing – review & editing. YC: Investigation, Methodology, Writing – original draft. DT: Conceptualization, Investigation, Methodology, Writing – original draft, Writing – review & editing. SS: Data curation, Formal analysis, Supervision, Validation, Writing – review & editing. TG: Data curation, Formal analysis, Writing – review & editing. XH: Software, Visualization, Writing – review & editing. YZ: Conceptualization, Funding acquisition, Project administration, Supervision, Writing – review & editing.
